# Liver transplantation in gastroenteropancreatic neuroendocrine tumors

**DOI:** 10.3389/fonc.2022.1001163

**Published:** 2023-02-09

**Authors:** Eduardo de Souza M. Fernandes, Camila V. Garcia Kyt, Felipe Pedreira Tavares de Mello, Leandro Savattone Pimentel, Ronaldo de Oliveira Andrade, Camila Girão, Camilla César, Munique Siqueira, Maria Eduarda Monachesi, Anderson Brito, Claudia Cristina Tavares de Sousa, Wellington Andraus, Orlando Jorge M. Torres

**Affiliations:** ^1^ Department of Gastrointestinal and Transplant Surgery, São Lucas-Rede Dasa Hospital, Rio de Janeiro, RJ, Brazil; ^2^ Department of Gastrointestinal and Transplant Surgery, Adventista Silvestre Hospital, Rio de Janeiro, RJ, Brazil; ^3^ Department of Surgery, Rio de Janeiro Federal University, Rio de Janeiro, RJ, Brazil; ^4^ Department of Hepatology, São Lucas-Rede Dasa Hospital, Rio de Janeiro, RJ, Brazil; ^5^ Department of Gastroenterology, Gastrointestinal and Transplant, São Paulo University Hospital, São Paulo, SP, Brazil; ^6^ Department of Hepatopancreatobiliary Surgery, Hospital São Domingos-Rede Dasa Hospital, São Luís, MA, Brazil; ^7^ Department of Gastrointestinal and Transplant Surgery, Hospital Presidente Dutra, São Luis, MA, Brazil

**Keywords:** neuroendocrine tumors, metastases, liver transplant, liver metastasis, transplant oncology, neuroendocrine cancer

## Abstract

Neuroendocrine tumors are part of a heterogeneous group of tumors located in organs such as the gastrointestinal tract (GIT), lungs, thymus, thyroid, and adrenal glands. The most prevalent sites are the small intestine, cecal appendix, and pancreas. More than 50% of these tumors are associated with metastases at the time of diagnosis. Neuroendocrine tumors are classified according to the degree of cell differentiation and the histopathological proliferation index of the lesion. Neuroendocrine tumors can be well differentiated or poorly differentiated. G3 tumors are characterized by Ki-67 expression greater than 20% and can be either well differentiated (G3 NET) or poorly differentiated (G3 NEC). Neuroendocrine carcinoma (NEC G3) is subdivided into small-cell and large-cell types. When neuroendocrine tumors present clinical and compressive symptoms, carcinoid syndrome is evident. Carcinoid syndrome occurs when the tumor produces neuroendocrine mediators that cannot be metabolized by the liver due to either the size of the tumor or their secretion by the liver itself. Several therapeutic strategies have been described for the treatment of metastatic neuroendocrine tumors, including curative or palliative surgical approaches, peptide receptor radionuclide therapy, percutaneous therapy, systemic chemotherapy, and radiotherapy. Liver surgery is the only approach that can offer a cure for metastatic patients. Liver metastases must be completely resected, and in this context, orthotopic liver transplantation has gained prominence for yielding very promising outcomes in selected cases. The aim of this study is to review the literature on OLT as a form of treatment with curative intent for patients with gastroenteropancreatic neuroendocrine tumors with liver metastasis.

## Introduction

Neuroendocrine tumors (NETs) originate from the neuroendocrine system, are part of a heterogeneous group of tumors widely distributed throughout the human body, and may be located in organs such as the gastrointestinal tract (GIT), lungs, thymus, thyroid, and adrenal glands. The most prevalent sites are located in the gastroenteropancreatic tract: the small intestine, cecal appendix, and pancreas (1).

NETs are a highly heterogeneous class of tumors in terms of clinical behavior, which complicates diagnosis and treatment (2).

Historical perceptions of NETs as indolent tumors are inadequate, as more than 50% of these tumors are associated with metastases at the time of diagnosis (1). In addition, exponential growth has been observed in the annual incidence of NETs. In 1973, the annual incidence of NETs in the United States was 1.31 per 100,000 inhabitants (3), while in 2003, this number rose to 2.47 per 100,000 inhabitants (4). More recently, the incidence of NETs in the United States was 6.98 per 100,000 inhabitants (5).

NETs are classified based on the degree of cell differentiation and the histopathological proliferation index of the lesion (**Table 1**). According to the most recent version of the classification according to the *National Comprehensive Cancer Network* (NCCN) (7) in 2021, NETs are classified according to the degree of cell differentiation and the histopathological proliferation index of the lesion. NETs can be well differentiated or poorly differentiated. G3 tumors are characterized by Ki-67 expression greater than 20% and can be either well differentiated (G3 NET) or poorly differentiated (G3 NEC). Neuroendocrine carcinoma (NEC G3) is subdivided into small-cell and large-cell types (8).

**Table 1:** Classification of Neuroendocrine Neoplasms (6).

Grade	Differentiation	Mitotic Index/Ki-67
1 (low) - NET	Well differentiated	< 2 mitoses/10 HPF < 3% Ki-67
2 (intermediate) - NET	Well differentiated	2-20 mitoses/10 HPF 3-20% Ki-67
3 (high) - NET	Well differentiated	> 20 mitoses/10 HPF > 20% Ki-67
Neuroendocrine carcinoma (NEC)	Poorly differentiated Small cells Large cells	> 20 mitoses/10 HPF > 20% Ki-67

NET, neuroendocrine tumor.

NETs originate from multipotent stem cells that progress to cancer due to genetic changes (inherited or acquired mutations). Depending on their genetic profiles and mutations, these multipotent cells may differentiate into epithelial, glandular, or neuroendocrine cancer cells. The most substantiated hypothesis, according to Rindi et al. (9), is that NETs and NEC have separate origins. However, another hypothesis is supported by a study by La Rosa et al. (10), which suggests that NETs may gradually progress to neuroendocrine carcinoma, but this hypothesis does not apply to most NET cases (11–13). Notably, NETs and NEC are genetically and clinically distinct (13), and NETs may be a primary liver cancer in very rare cases.

Most patients with NETs are asymptomatic, even when they have metastasis. When they present clinical symptoms, compressive symptoms and carcinoid syndrome are evident. Carcinoid syndrome occurs when the tumor produces neuroendocrine mediators (serotonin, corticotropin, histamine, and dopamine, among others), which cannot be metabolized by the liver due to either the size of the tumor or their secretion by the liver itself, thus precluding the first-pass effect. These cases clinically present with facial flushing (70 to 90%), abdominal pain, diarrhea, pellagra, bronchospasm, and heart valve disease (14).

Several therapeutic strategies have been described for the treatment of metastatic NETs, including curative or palliative surgical approaches, peptide receptor radionuclide therapy, transarterial therapy, percutaneous therapy, systemic chemotherapy, biotherapy, and radiotherapy (15–18). Palliative approaches include debulking for uncontrolled functioning syndrome and surgical resection of the primary tumor. Ablative locoregional and vascular treatments for G1-G2 NETs are predominantly employed in cases where disease is confined to the liver or cases with stable extrahepatic disease. This approach is indicated for the relief of symptoms due to hormone secretion or the mass effect and aims to improve quality of life. Liver-directed therapies for metastatic NETs include transarterial embolization (TAE), thermal ablation, transarterial chemoembolization (TACE), and transarterial radioembolization (TARE), also known as selective internal radiation therapy (SIRT).

Liver surgery is the only approach that can offer a cure for patients with metastatic disease. The resection of accessible tumor deposits in the liver is regarded as the standard treatment and improves survival. However, NETs have strong metastatic potential, which results in a high tumor recurrence rate and consequently makes achieving a cure in this group of patients difficult (17, 19). In addition, micrometastases are already present in the liver in many cases and are not identifiable on imaging tests, resulting in a high rate of tumor recurrence after surgical resection (20). Because the primary lesion and locoregional metastases must be completely resected, a radical approach with total resection of the metastatic liver in the context of orthotopic liver transplantation (OLT) has gained prominence for yielding very promising outcomes in selected cases (16).

This study aims to review the literature on OLT as a form of treatment with curative intent for patients with gastroenteropancreatic NET with liver metastasis (NET-LM).

## Transplant oncology

The two indications for OLT for oncological diseases are primary malignant liver cancer and liver metastasis. Hepatocellular carcinoma (HCC) is a disease for which OLT is well established as a form of treatment (21). Liver metastasis of colorectal cancer has also shown promising results after OLT, with improved overall and disease-free survival rates (22, 23).

NETs may be a primary liver cancer in very rare cases (24). If this tumor is not resectable and in the absence of extrahepatic disease, liver transplantation may be a therapeutic option. With respect to NET-LM, hepatectomy with broad lymphadenectomy followed by OLT theoretically allows the best oncological resection of hepatobiliary malignancies; however, at least two issues limit the acceptance and applicability of transplantation as the first line of treatment. The first issue is the importance of weighing the risks and benefits of performing a transplant considering overall survival with immunosuppression and the risk of developing disease recurrence in immunosuppressed patients. The second issue is the continuous scarcity of organ donors, which places patients at risk of tumor progression while on the waiting list for transplantation (16). The time waiting for an organ competes with the desire to resect lesions.

Transplantation for the treatment of NET-LM involves the use of liver grafts from cadaver donors, grafts from living donors, or multivisceral transplantation (MVT) depending on indication criteria (23).

## Patient selection for orthotopic liver transplantation

Several liver transplantation programs have published criteria for the selection of NET-LM patients; however, two such criteria have gained popularity among surgeons worldwide (**Table 2**) (25, 26). The Milan criterion was developed in 1995 by the National Cancer Institute of Milan and was revised in 2016 with the publication by Mazzaferro et al.; among 280 patients analyzed in the retrospective study from 1995 to 2010, 88 were eligible for transplantation after being identified according to the selection criteria. They divided the patients into those undergoing OLT (26) and the control group, which included patients for whom OLT was not performed (27). At 5 years, the overall survival and disease-free survival rates were 97 and 89% among the OLT patients, respectively, while the control group had a 5-year survival rate of 50.9%. The indication criteria for transplantation were based on having fewer risk factors for tumor recurrence, as confirmed by the good results obtained (2, 28, 29). The UNOS (*United Network for Organ Sharing)* criterion contains the prerequisites of the Milan criterion and adds other criteria based on tumor recurrence control in an attempt to reduce the risk of transplantation waiting list dropout. The UNOS guidelines postulate the need for the absence of tumor recurrence for 3 months and add that even when positive findings for lymph node metastasis are uncovered, such findings must become negative for at least 6 months before re-enlisting the patient. In addition, the UNOS requires a positron emission tomography (PET) scan since it is the gold standard test for diagnosis (30).

**Table 2:** Selection criteria for orthotopic liver transplantation for liver metastasis of a neuroendocrine tumor.

Selection criteria of Milan-NET (2007, revised in 2016)	
1) Absolute - G1 or G2 histological grade - Primary tumor with portal drainage - Exclusion of any extrahepatic disease - Tumor liver invasion < 50% - Stable disease > 6 months
2) Relative - Age < 60=““>
Guideline recommendation of the UNOS for orthotopic liver transplantation for liver metastasis	
1) Common criteria with Milan-NET - Histological grade G1 or G2 - Tumor of gastroenteropancreatic origin with drainage through the portal system - Primary and extrahepatic resection without recurrence > 6 months - Tumor extension < 50% of the liver volume - Age of the recipient < 60 years
2) Additional criteria - Unresectable liver metastasis - Radiographic characteristics of NET-LM - Negative PET scan for metastasis activity - Absence of tumor recurrence > 3 months - In the presence of positive findings for lymph node metastasis on a PET scan, the findings should become negative at least 6 months before re-enlisting - In the presence of extrahepatic metastasis in a solid organ (i.e., lung or bone), the case will be permanently unlisted

## Diagnostic criteria

When approving OLT as a treatment modality for these patients, a detailed clinical evaluation, functional status evaluation, and detailed radiological evaluation of the disease must be performed. In terms of the radiological evaluation, computed tomography of the abdomen should include an evaluation of the arterial phase, since most of these metastases are hypervascularized (31). Diffusion-weighted magnetic resonance imaging (DW-MRI) plays an essential role and should be performed systematically because it captures lesions smaller than 1 cm (32). The gold standard imaging technique for the disease is PET, which can display functional images. For G1 or G2 tumors, PET should be performed with ^68^Ga-radiolabeled DOTA peptides, which can detect lesions that other examination modalities, such as somatostatin-receptor scintigraphy with indium-111-conjugated radiopharmaceutical, do not detect (33). PET provides relevant data to accurately determine the surgical indication, since it has a sensitivity of 82-100% and a specificity of 67-100% and identifies extrahepatic disease with a sensitivity of 85-100% and a specificity of 67-100%. ^68^Ga-DOTA PET is especially relevant when transplantation is an option because it better selects candidates and excludes patients with extrahepatic metastasis (34), as demonstrated in a study in which ^68^Ga-DOTA PET changed the therapeutic strategy for approximately 33% of patients (35). On the other hand, tumors with an intermediate or a high degree of differentiation, especially with Ki-67 > 15%, seem to be better evaluated using FDG PET, with a sensitivity of 92% versus 69% compared to ^68^Ga-DOTA PET (36).

## Literature review

In the 21st century, several publications on OLT as a form of NET-LM treatment have gained prominence. We highlight studies with more than 10 patients, including prospective and retrospective studies and multicenter and single-center studies. Notably, the studies used different indication criteria and different preoperative preparations, which resulted in different outcomes.

The robust study by Mazzaferro et al. (2) in 2016 prospectively analyzed controlled and nonrandomized cases of NET-LM from 1995 to 2010. After some inclusion and exclusion criteria were applied, this study compared 42 transplant patients with 46 patients in the control group who did not receive transplants. The indication criterion used was Milan-NET 2016. The transplanted group had survival rates of 97.2% and 88.8% at 5 and 10 years, respectively, while the control group had half of the survival rate at 5 years and one-fourth of the survival rate at 10 years. The two groups analyzed had important differences. The transplant patients were younger, received more locoregional than systemic treatment, and had a lower tumor grade and tumor stage, resulting in a possible selection bias. On the other hand, 88% of the transplant patients received marginal liver grafts, with a mean of 40% steatosis and 8 hours of cold ischemia (2). Using the appropriate principle of marginal organ graft use, a recipient’s good clinical condition improves results in the medium and long term with this technique (37). This study strengthens the indication of the Milan-NET criteria, as it demonstrates a considerable long-term benefit in terms of survival. The most interesting finding presented by Mazzaferro is that the benefit of OLT increases over time, since the survival gains in favor of transplantation compared to no transplantation were 6.8 months at 5 years and 38.4 months at 10 years.

In France in 2013, Le Treut (38) led a multicenter study with 213 patients from 35 centers in Europe undergoing transplantation from 1982 to 2009. The 5-year survival rate was 52%. This publication indicates the factors that correspond to a worse prognosis: hepatomegaly, upper-abdominal exenteration, and tumors with poor differentiation. However, this study included not only transplant patients because of oncological indications with curative intent but also patients with hormonal syndrome, tumor debulking, and iatrogenic complications, including primary tumors drained through the cava system, which may have led to underestimated overall survival in the study (39). Only 38 of the last 106 cases (between 2000 and 2009) were subjected to the Milan-NET criterion, and rates of 79% for overall survival and 51% for 5-year survival were obtained (38). Additionally, OLT should not be associated with major extrahepatic resection, and the primary tumor should not be removed at the same time as liver transplantation, especially if the primary tumor is in the pancreatic head. These data were reinforced years later by Lim et al. (40) in 2018 in their literature review, highlighting that in addition to reducing surgical morbidity, resecting the primary tumor before transplantation is beneficial because it allows better access to tumor biology and consequently a better understanding of the prognostic factors that will determine the surgical indication (40). Notably, OLT should no longer be indicated with palliative intent. As written by Lim et al. when analyzing the ELTR study, transplantation with palliative intention may be beneficial, but the result is discouraging because the mean survival was 20 months and the 5-year survival rate was 15% for resection margins of R1 (13 cases) or R2 (seven cases) (17, 38, 41).

In terms of age as a selection criterion for OLT, several authors suggest that an age greater than 60 years is a risk factor for a negative outcome (2, 17, 38). However, Sher et al. note that age is not a prognostic factor for a negative outcome, whereas the invasion of large vessels is a factor (42). Nevertheless, transplantation for patients younger than 60 years remains strongly recommended, as corroborated by the abovementioned criteria.

## Primary pancreatic tumors

Primary pancreatic NETs (pNETs) have shown alarming results compared to those for other sites in which NETs occur with respect to tumor recurrence and survival. An ELTR analysis evaluating 213 patients highlights that pNET patients have worse 5-year survival than patients with primary tumors in other sites of the GIT. In 40% of patients transplanted due to pNETs, the primary cause of death was tumor recurrence, with a mean survival of 52 months after transplantation.

A Hungarian group led by Korda et al. published a cohort study analyzing 10 patients undergoing OLT due to NET-LM. The study does not discuss the indication criteria used, but it states that the overall 1-year and 5-year survival rates were 89% and 71%, respectively. However, the study reinforces the idea that tumor recurrence is worse in pNET patients. Of the 10 analyzed patients, all three patients in whom the primary tumor was located in the pancreas relapsed (43).

These data corroborate the findings of Van Vilsteren et al. (44), whose retrospective analysis of operated cases according to the current UNOS criterion showed that among the 19 operated patients and with a complete follow-up, 11 had pNETs, all of which had high 5-HIAA levels. Among all 19 patients, three experienced tumor recurrence, all of whom had pNETs (25). In addition, evidence of relapse was also identified, and all had Ki-67 levels greater than 5%, suggesting that the mitotic index interferes with relapse, as stated by several authors (17, 42). These data are reinforced when analyzing the recent publication by Moradi et al. (45) from 2021, where four patients were transplanted due to pNETs, but three died early (2 to 5 months) due to primary graft dysfunction and multiorgan failure.

## Waiting list

Classically, eligibility for the waiting list for OLT, regardless of the cause, is determined by the *Model for End-Stage Liver Disease* (MELD) score, which was introduced in 2002. As OLT candidates with NET-LM have preserved liver function, the MELD score is low. Therefore, special scores must be acquired to realize the possibility of receiving a graft. Therefore, a special score is applied to the patient profile for proper allocation to the transplant waiting list (27). Currently, the applicability of the special score is not homogeneous. Because NET-LM is a rare condition with a very strict surgical indication with respect to OLT, NETs do not impact the transplant waiting list.

Although OLT is the main strategy to cure this group of patients, Nobel et al. (46) disagreed with this opinion in 2015. This study, which analyzed data published in the UNOS from 240 patients correlating MELD and outcomes, suggests that this nonstandard process of assigning special scores may improve waiting list prioritization for patients whose posttransplant survival results are lower than those of the others listed. The study indicates that restricting transplantation to those with acceptable posttransplantation results should be considered, although more prospective studies must be conducted to reach this conclusion.

## Prognostic factors

A better understanding of the relevant prognostic factors may contribute to better selection of patients who are candidates for OLT due to NET-LM. Among the possible locations for a primary tumor to settle, a tumor in the pancreas has a worse prognosis, as identified by several groups. The largest cohort of patients who received OLT to treat NET-LM (213 patients), which was published in 2013 by Le Treut, analyzed multicenter data from the ELTR (*European Liver Transplant Registry)* and revealed three main independent predictors of a negative outcome: major resection associated with OLT, a poorly differentiated tumor, and hepatomegaly (defined by an increase > 25% in the volume of the explanted liver in relation to the predicted liver volume) (38).

In 2015, another multicenter analysis published by Sher et al. (26) revealed the factors associated with a worse outcome: a poorly differentiated tumor, transplants performed before the 2000s (pre-MELD era), significant vascular invasion, and surgical procedures combined with OLT, such as multivisceral exenteration, pancreatoduodenectomy, and distal pancreatectomy. Another multicenter study of 240 patients by Nobel et al. strongly suggested that a serum bilirubin level greater than 1.3 mg/dl can be used as a prognostic factor because outcomes substantially differed by bilirubin status. Specifically, bilirubin > 1.3 mg/dl corresponded to a worse prognosis. The group of patients with serum bilirubin > 1.3 mg/dl had 1-year and 3-year survival rates of 70.8% and 36.4%, respectively, compared to 91.3% and 78.3% in the group with serum bilirubin < 1.3 mg/dl (46).

The risk factors for recurrence usually considered during patient selection for OLT to treat NET-LM are listed in **Table 3**. These and other factors confirm that the management of NET-LM must be individualized and evaluated in a multidisciplinary manner (6, 33).

**Table 3:** Risk Factors for Tumor Recurrence after Orthotopic Liver Transplantation (OLT) (19).

1. Age > 50 years	
2. Tumor burden and symptomatic control as surgical indication	
3. Primary neuroendocrine tumor of the pancreas	
4. Primary tumor with drainage outside the portal system	
5. Noncarcinoid tumor	
6. Ki-67 > 10%	
7. Aberrant E-cadherin expression	
8. Invasion of large vessels	
9. Liver involvement > 50% of the predicted liver volume	
10. Primary tumor not resected before transplantation	
11. Extrahepatic lymph node invasion	
12. Tumor recurrence at < 6 months of clinical observation	
13. Poorly differentiated neuroendocrine tumor	
14. Major extrahepatic resection	

## Waiting time for OLT among NET-LM patients

Another point of considerable controversy is the ideal time to indicate OLT in this group of patients. Several authors argue that OLT should be indicated for patients who respond to systemic treatment or who have stable disease for at least 6 months. Others have considered OLT for patients with systemic drug therapy refractoriness and the presence of progressive liver disease. The current trend suggests that patients should be subjected to a clinical and radiological observation period to better assess the tumor biology and define the aggressiveness of the tumor after resection of the primary tumor. Analysis of the UNOS data indicated better outcomes for patients who were under observation for more than 2 months before undergoing OLT.

An analysis of the ELTR data in 2013 reveals that the time between NET-LM diagnosis and OLT and the number of patients undergoing surgical procedures and locoregional therapies before OLT increased after 2000. However, the time between diagnosis and OLT did not interfere with patient outcomes. Notably, during this observation period, systemic therapies, such as somatostatin analogs or targeted therapy, are reasonable to control symptoms and tumor growth (20).

## Conclusion

Liver transplantation for unresectable and liver-restricted NET is an interesting therapeutic alternative and has achieved excellent results in recent studies. A multidisciplinary evaluation is important when selecting both patients and the best therapeutic strategy. In addition, recurrence, which is common, can be treated after transplantation with good survival.

## Author contributions

All authors listed have made a substantial, direct, and intellectual contribution to the work and approved it for publication.
